# Exposure to Traffic-Related Particulate Matter 2.5 Triggers Th2-Dominant Ocular Immune Response in a Murine Model

**DOI:** 10.3390/ijerph17082965

**Published:** 2020-04-24

**Authors:** Hyun Soo Lee, Sehyun Han, Jeong-Won Seo, Ki-Joon Jeon

**Affiliations:** 1 Department of Ophthalmology, Eunpyeong St. Mary’s Hospital, College of Medicine, The Catholic University of Korea, Seoul 03312, Korea; 2 Department of Environmental Engineering, Inha University, Incheon 22212, Korea; shhaninhauniv@gmail.com (S.H.); kjjeoninha@gmail.com (K.-J.J.); 3 Department of Ophthalmology, Hallym University Dongtan Sacred Heart Hospital, College of Medicine, Hallym University, Gyeonggi-do 18450, Korea; jwseohallym@gmail.com

**Keywords:** air pollution, allergic conjunctivitis, dendritic cell (DC), dry eye disease (DED), particulate matter (PM)

## Abstract

Ambient particulate matter (PM), a major component of air pollution, aggravates ocular discomfort and inflammation, similarly to dry eye disease (DED) or allergies. However, the mechanism(s) by which PM induces the ocular inflammatory response is unknown. This study investigated the immunological response of traffic-related fine particulate matter (PM_2.5_) on the ocular surface in a murine model. C57BL/6 mice were exposed by topical application to PM_2.5_ or vehicle for 14 days to induce experimental environmental ocular disease. Corneal fluorescein staining and the number of ocular inflammatory cells were assessed in both groups. The expression of IL-1β, IL-6, tumor necrosis factor (TNF)-α, and mucin 5AC (MUC5AC) in the ocular surface were evaluated by real-time PCR. An immunohistochemical assay evaluated apoptosis and goblet cell density. ELISA was used to determine the levels of serum IgE and cytokines of Type 1 helper (Th1) and Type 2 helper (Th2) cells after in vitro stimulation of T cells in the draining lymph nodes (LNs). Exposure to traffic-related PM_2.5_ significantly increased corneal fluorescein staining and cellular toxicity in the corneal epithelium compared with the vehicle control. A significant increase in the number of CD11b+ cells on the central cornea and mast cells in the conjunctiva was observed in the PM_2.5_ group. Exposure to PM_2.5_ was associated with a significant increase in the corneal or conjunctival expression of IL-1β, IL-6, TNF, and MUC5AC compared to the vehicle, and increased maturation of dendric cells (DCs) (MHC-II^high^CD11c^+^) in draining LNs. In addition, PM_2.5_ exposure increased the level of serum IgE and Th2 cytokine production in draining LNs on day 14. In conclusion, exposure to traffic-related PM_2.5_ caused ocular surface damage and inflammation, which induced DC maturation and the Th2-cell-dominant allergic immune response in draining LNs.

## 1. Introduction

Due to industrialization, air pollution has gained attention as a major risk factor for respiratory and cardiovascular diseases, with increased morbidity and mortality worldwide [1,2,3]. With continued urbanization, air-pollution-related health problems are expected to worsen with time [1,2]. Sources of air pollution include combustion of wood and fossil fuels, road transport, and building construction, which can be organized into carbon, heavy metals, nitrates, sulfates, ozone, polycyclic aromatic hydrocarbons, and toxic pollutants. These air pollutants vary by size, composition, and source [3,4,5]. Particulate matter (PM) is one of the major components of air pollution, and is used to indicate the severity of air pollution [5,6]. Recent studies have focused on fine particles (PM_2.5_; particulate matter less than 2.5 μm in aerodynamic diameter) in air pollution, which are more likely to have adverse medical effects than larger particles [6,7]. PM_2.5_ is harmful to the respiratory and cardiovascular systems, causing asthma, vascular ischemia, and thrombosis [4,5,6,7]. 

The eyes are very sensitive to environmental agents because they are protected from harmful matter by only a thin layer of tear film [8]. Various studies suggest that air pollution could cause ocular irritation and subclinical inflammation, which is associated with dry eye disease (DED) or allergic conjunctivitis [9,10,11,12]. Recently, studies on the correlation between ocular symptoms and levels of PM have been gaining attention [9,10,11]. However, the mechanisms by which PM affects the eyes have not been sufficiently elucidated. Most research has investigated the influence of diesel exhaust particles or titanium dioxide on eye disease [13,14,15], which does not accurately reflect the characteristics of PM in the eyes. Therefore, it is necessary to conduct research on the effects of PM in the eyes and ocular diseases using traffic-related PM from actual road dust. Research by Karagulian et al. found that 25% of ambient PM was from traffic, more than industry, domestic fuel burning, and undetermined sources of human origin [16]. The main sources of traffic-derived PM_2.5_ were engine emissions, road dust, and tire wear [9,16]. 

However, although air pollution, including PM, is associated with ocular diseases such as dry eye and conjunctivitis, the mechanism by which traffic-related PM_2.5_ causes environmental ocular disease has not been extensively studied [9,17,18]. Therefore, we used traffic-related PM_2.5_ collected from road dust to determine how PM alters the immune response in the healthy mice.

## 2. Materials and Methods

### 2.1. Animals

Eight to nine week old male C57BL/6 mice (Charles River Laboratories, Orient Co., Sungnam, Korea) were housed in pathogen-free conditions at the Catholic Medical Institute Animal Care Facility. All procedures and protocols (ethical code: 2019-0261-01) were approved by the Catholic Medical Research Institute Animal Care and Use Committee, and all animals were treated according to the Statement for the Use of Animals in Ophthalmic and Visual Research (ARVO). Mice received a topical application of 4 μL of 0.5mg/mL traffic-related PM_2.5_ twice daily for 14 days. Age- and sex-matched mice were used as the normal controls. After 14 days, ocular injury was evaluated by corneal fluorescein staining. First, 1 μL of 1% fluorescein (Sigma-Aldrich) was administrated to the inferior–lateral conjunctival sac of the eye. After three minutes, corneal fluorescein staining was evaluated using a slit lamp biomicroscope. Punctate corneal staining was calculated in a masked fashion using the National Eye Institute grading system, which presents a score of 0 to 3 for each of the five areas of the cornea [19].

### 2.2. Collection and Recovery of Traffic-Related PM_2.5_

Road dust was collected using a vacuum sweeper from a heavy traffic zone in Gangdong-Gu, Seoul, Korea, according to our previous procedures [20]. After baking the collected samples at 100 °C and sieving the particulate matter (PM) less than 75 μm, the samples were then suspended to collect PM_2.5_, which has a diameter of 2.5 μm or less, using a Deakti Impactor (Dekati Ltd., Kangasala, Finland). This PM_2.5_ sample was mixed with phosphate-buffered saline (PBS) for 15 min under a sonication water bath. The total PM suspension was diluted with PBS to a concentration of 0.5 mg/mL [15,21]. Lipopolysaccharide levels were quantitated using a LAL Chromogenic Endotoxin Assay (Thermo Scientific, Waltham, Massachusetts, USA) and were below < 0.1 EU/mL, as the limits of detection. The mice were divided into two groups, a vehicle (PBS) control group, and the PM_2.5_ challenge group.

### 2.3. RNA Isolation and Real-Time Polymerase Chain Reaction 

We determined the cytokine expression profiles in the ocular surface. Isolation and purification of total RNA from the corneal and conjunctival tissue was done using Trizol (Invitrogen) and RNeasy Microkit (Qiagen). Complementary DNA (cDNA) was synthesized using SuperScript III^TM^ reverse transcriptase (Invitrogen), and a quantitative real-time polymerase chain reaction was carried out using Taqman Universal PCR Mastermix and FAM-MGB dye-labeled primers (Applied Biosystems) for IL-1β (Mm00434228_m1), IL-6 (Mm00446190_m1), TNF (Mm99999068_m1), mucin 5AC (MUC5AC) (Mm 01276705_g1), and glyceraldehydes 3-phosphate dehydrogenase (GAPDH) (Mm99999915_g1). One microliter of cDNA was put into each well in duplicate. The GAPDH gene was used as the endogenous reference for each reaction. Real-time PCR data were analyzed by the comparative threshold cycle method, and the relative expression level of each sample was presented as a fold change from normal control.

### 2.4. CD11b-Positive Cellular Infiltration by Immunohistochemical Staining

Fluorescein isothiocyanate (FITC)–conjugated rat anti–mouse CD11b (1:100; monocyte/macrophage marker; Biolegend, CA) and FITC-conjugated rat IgG2bk (isotype control; Biolegend) were used for the immunohistochemical assay. Three corneas from three mice per group were used and dissected corneas were fixed with acetone for 15 min at Day 14, as previously described [22,23]. After blocking nonspecific staining with an anti-FcR CD16/CD32 antibody (Biolegend), the specimens were immunostained with primary or isotype antibodies overnight, washed three times with PBS, incubated with secondary antibodies, and mounted with Vector Shield with DAPI (4,6 diamidino-2-phenylindole, Vector Laboratories). CD11b^+^ cells were counted at two areas in the center (within 2 μm of the center) of each cornea in a masked fashion under an epifluorescence microscope (Nikon E800) at 40× magnification. The mean number of cells was analyzed by averaging the cell number in each area. The data are presented as averages ± SEM of all mice observed.

### 2.5. Periodic Acid Schiff (PAS) Staining for Conjunctival Goblet Cells

Ipsilateral whole eyeballs, including the superior and inferior forniceal conjunctiva, were excised from PM_2.5_-treated or vehicle control mice following topical challenges for 14 days. Cryosections (7 μm) at the center of the eye were air-dried for 30 min and subjected to periodic acid Schiff (PAS) staining. To count goblet cells, the average number of PAS-stained cells on the four different sections from each eye was calculated under a microscope (Eclipse E400, Nikon, Melville, NY, USA) using a ×20 objective by two observers in a blind study.

### 2.6. Single-Cell Isolation from Draining Lymph Nodes and Conjunctivae For Flow Cytometric Analysis

Single cells from conjunctivae and draining LNs were prepared with a 70 μm cell strainer. Cellular viability was confirmed by Trypan blue exclusion assay. After incubating with Fc-blocking antibody in 0.5% BSA at 4 °C for 30 min, and the cells were immunostained with FITC-conjugated anti-CD45, PE-Cy7-conjugated anti-CD11c, PE-Cy7-conjugated anti-c-kit, or PE-conjugated anti-I-A^b^. Isotype control was used with the relevant antibodies (eBioscience). Stained cells were acquired on a BD™ LSR II flow cytometer (Becton-Dickinson, Franklin Lakes, NJ, USA) and analyzed with Flowjo software (FlowJo, Ashland, OR, USA).

### 2.7. In Vitro Stimulation of T Cells

This procedure has been previously described [24]. Cervical draining LNs were harvested from neck dissection and T cells were isolated via magnetic-bead sorting using anti-CD90.2 antibodies (Miltenyi Biotec, Bergisch Gladbach, Germany). Enriched T cells were loaded into 96 well plates at a concentration of 1.5 × 10^6^/mL. Bone-marrow-related dendritic cells (BMDCs) prepared as previously described were co-cultured with T cells (0.75 × 10^6^/mL) and 10% FBS for 48 h. The supernatants were harvested after stimulation with PMA/ionomycin (Sigma-Aldrich Corp., St. Louis, MO, USA) for 6 h. Cytokines such as IFN-Ɣ, IL-4, and IL-13 were measured via ELISA, as per manufacturer’s instructions (Ready-set-go ELISA kit; eBioscience, Waltham, MA, USA).

### 2.8. Quantitation of IgE in Serum

Following 14 days of topical PM_2.5_ challenge, blood was collected by cardiac puncture after euthanasia. Sera were isolated using coagulation and centrifugation, and then analyzed using an ELISA kit for total mouse IgE (88-50460, eBioscience, Waltham, MA, USA).

### 2.9. TdT-Mediated dUTP Nick End Labeling (TUNEL) Assay

We evaluated surface epithelial damage using a TdT-mediated dUTP nick end labeling (TUNEL) assay. To evaluate corneal epithelial cell damages, 7 μm cryostat cross-sections were fixed in 4% paraformaldehyde at Day 14, and TUNEL staining was then performed according to the manufacturer’s protocol (TUNEL Kit, Roche, Basel, Switzerland). Images were obtained under an epifluorescence microscope with 100× magnification. Both TUNEL-positive and DAPI-positive cells were calculated at the central cornea (100 μm width × 40 μm depth areas of epithelial layer) in a masked fashion, as previously described [25]. 

### 2.10. Statistical Analysis

Data are expressed as mean ± standard error of the mean (SEM) of three independently repeated experiments. Statistical significance among the groups was analyzed via a one-way ANOVA followed by Tukey’s post hoc tests using Prism software (version 5.0; GraphPad, San Diego, CA, USA). *p* < 0.05 was considered statistically significant.

## 3. Results

### 3.1. Clinical Signs of Ocular Surface Injury 

**Figure 1:**
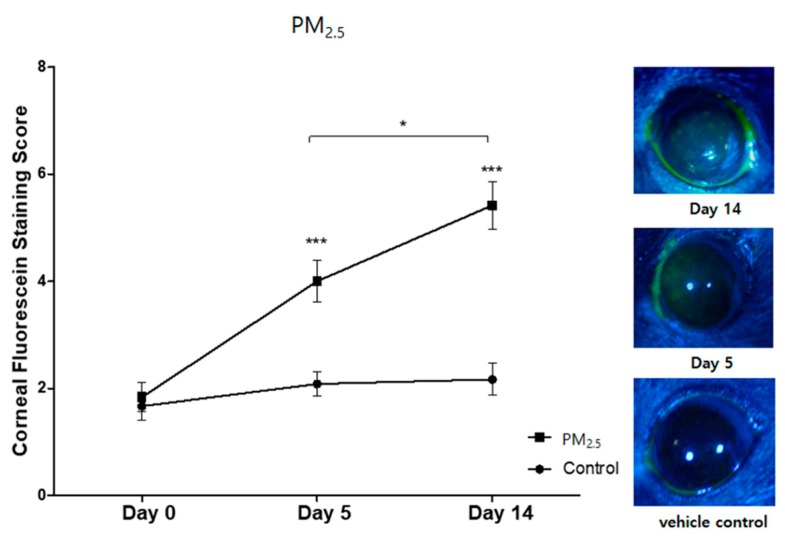
Traffic-related PM_2.5_ challenges led to a significant increase in the corneal fluorescein staining score compared to the vehicle control at Day 5 and Day 14. * *p <* 0.05, *** indicates *p <* 0.0001. Data are presented as the mean ± standard error of the mean (SEM) of three experiments. Each experiment consisted of eight mice per group.

PM_2.5_ was applied to the mice for 14 days. At Days 5 and 14, PM-exposed groups showed a significant increase in corneal fluorescein staining compared to the vehicle control groups (*p* < 0.001, Figure 1).

### 3.2. Inflammatory Cells’ Infiltration to Cornea and Conjunctiva

**Figure 2:**
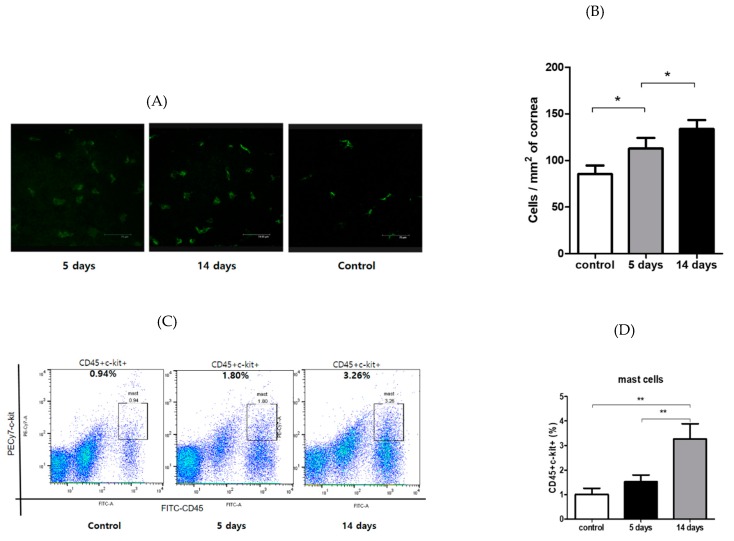
Inflammatory cells’ accumulation in the ocular surface in a murine model of traffic-related PM_2.5_ exposure. (**A**) Representative epifluorescence images showing CD11b+ cells (green) in the central corneas (scale bar: 75 μm). (**B**) Traffic-related PM_2.5_ increased the number of CD11b+ cells in the central corneas, compared to the vehicle-treated groups. (**C**) Conjunctivae were harvested from these mice and the flow cytometry of mast cells (CD45+c-kit+) was analyzed. Figures represent three independent experiments. (**D**) Data are representative of three independent experiments. *p*-value signs indicate: * *p <* 0.05, ** *p <* 0.001. Data are presented as the mean ± SEM of three experiments. Each experiment consisted of three to four corneas per group.

The mean number of corneal CD11b+ cells was 85.3 ± 4.6 cells/mm^2^ in the vehicle control group, and 112.7 ± 5.7 cells/mm^2^ and 133.6 ± 4.8 cells/mm^2^ in the PM_2.5_ challenge group at Day 5 and Day 14, respectively (*p* < 0.05, Figure 2A,B). Conjunctivae were also harvested for flow cytometric enumeration of mast cells (CD45+ c-Kit+). This population was augmented in PM_2.5_-exposed mice relative to the control group (*p* < 0.05, Figure 2C,D). These findings suggest that PM_2.5_ induced an allergic ocular response, as the mast cell is a key immune factor in the Th2-mediated responses. After the PM_2.5_ challenge, the degree of inflammatory cell infiltration at the ocular surface increased significantly compared to the vehicle control. 

### 3.3. Inflammatory Cytokine Expression in the Ocular Surface

**Figure 3:**
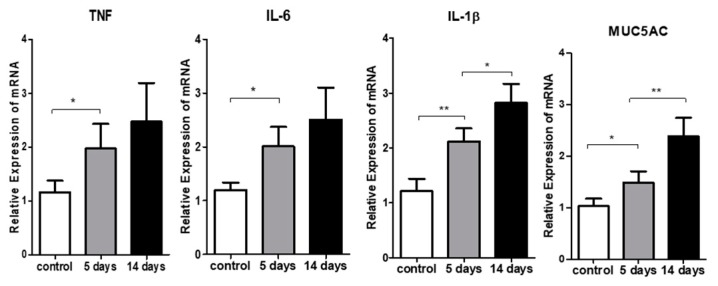
Cytokine expression in the ocular surface. Real-time polymerase chain reaction analysis showed that PM_2.5_ exposure significantly increased the relative expressions of TNF (* *p <* 0.05), IL-6 (* *p <* 0.05), and IL-1ß (* *p <* 0.05 and ^**^ *p <* 0.01) in the corneas, and mucin 5AC (MUC5AC) (* *p* < 0.05 and ^**^ *p* < 0.01) in the conjunctivae, compared to the vehicle control groups. Data were normalized to GAPDH mRNA as an internal control, and values were then expressed as the fold change over the normal naive corneas. Data are presented as the mean ± SEM of three experiments. Each experiment consisted of three corneas or conjunctivae per group.

**Figure 4:**
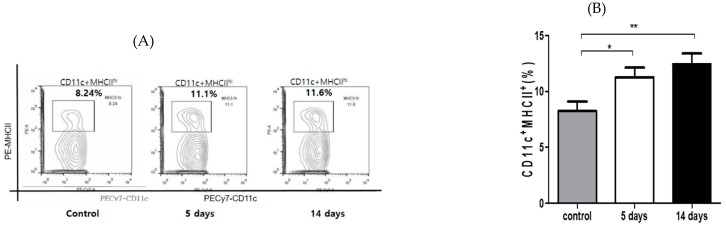
Ocular inflammation via exposure to traffic-related PM was associated with increased levels of mature dendritic cells (MHC-II^high^ CD11c^+^ cells) in the draining cervical lymph nodes (LN). (**A**) Representative flow data demonstrated that PM_2.5_ challenge increased the frequency of mature APCs (MHC-II^high^CD11c^+^) in the draining LN compared with the vehicle control. (**B**) Data are presented as the mean ± SEM of three independent experiments. Each experiment consisted of two mice per group. *p*-value signs indicate * *p* < 0.05; ** *p* < 0.001.

A real-time polymerase chain reaction was used to quantify the transcripts encoding IL-1β, IL-6, and TNF in the corneas and conjunctivae of the two groups (Figure 3). The PM_2.5_ challenge significantly increased relative expression of IL-1β (*p* <0.05 vs. vehicle), IL-6 (*p* < 0.05 vs. vehicle), and TNF-α (*p* < 0.05 vs. vehicle) transcripts at the corneas, and MUC5AC transcripts at the conjunctivae (*p* < 0.05 vs. vehicle). To explore the immunogenic function of antigen-presenting cells (APCs) in this model, we evaluated the frequencies of mature APCs (MHC-II^high^ CD11c^+^ cells) in the draining lymph nodes. PM_2.5_ exposure led to the maturation of APCs in draining LNs as compared to the vehicle controls (*p* < 0.05, Figure 4A,B).

### 3.4. Effect of PM_2.5_ on Apoptosis of the Corneal Epithelial Cells 

**Figure 5:**
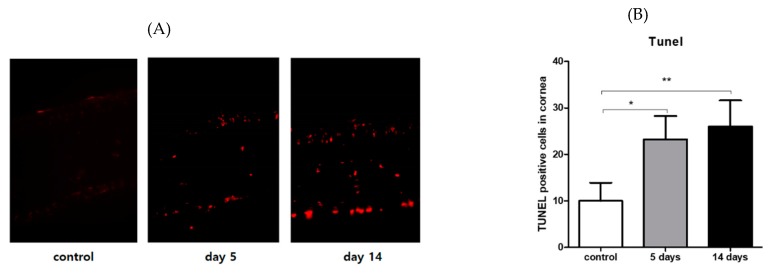
Quantitation of TUNEL-positive cells in the corneal epithelial layer. (**A**) Representative photograph of TUNEL staining of the cornea in both groups. (**B**) The number of apoptotic cells (red) in PM_2.5_-exposed corneas was significantly increased as compared to the vehicle control. *p*-value signs indicate * *p* < 0.05; ** *p* < 0.001. Data are presented as the mean ± SEM of three experiments. Each experiment consisted of three to four corneas per group.

The TUNEL assay indicated that apoptosis was induced in the superficial and basal epithelium of PM_2.5_-exposed corneas, whereas a few apoptotic cells were observed in the corneal epithelium of the vehicle group. The number of apoptotic cells was significantly increased in the PM_2.5_-exposed corneas at Days 5 and 14 compared with the vehicle controls (*p* < 0.005, Figure 5A,B). 

### 3.5. PM_2.5_ Did Not Decrease the Number of Goblet Cells in the Conjunctiva 

**Figure 6:**
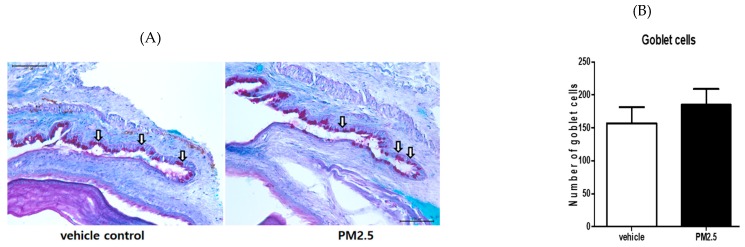
Conjunctival goblet cell density after PM_2.5_ exposure. (**A**) Representative photograph of periodic acid Schiff (PAS) staining of the conjunctiva at Day 14. (**B**) The goblet cell counts (white arrow) in the conjunctiva were not different between PM_2.5_ and vehicle control mice. The data are presented as the mean ± SEM. Each experiment consisted of four eyes per group. The scale bar indicates 100 μm.

PAS staining of the conjunctiva showed the number of goblet cells, which are responsible for mucous tear production. We measured the number of mucin-filled goblet cells in PAS-stained conjunctival sections. There was no statistically significant difference in the conjunctival goblet cells between the PM_2.5_ exposure group and the vehicle control group (*p* = 0.152, Figure 6A,B). 

### 3.6. PM_2.5_ Induced Type 2 CD4 T-Cell Immune Responses in the Draining LNs

**Figure 7:**
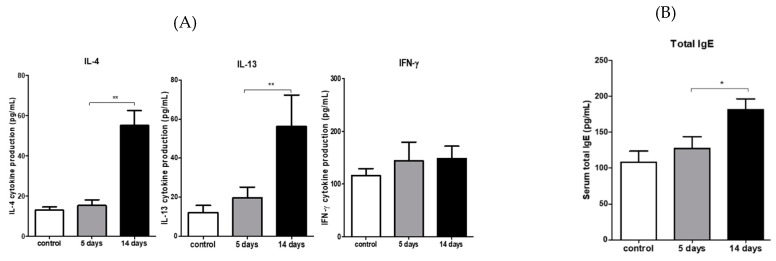
Traffic-related PM_2.5_ enhances Th2-mediated allergic responses in the eyes. (**A**) Draining cervical lymph nodes were harvested after the euthanasia of mice on Day 14 (n = four mice per group). Purified T cells with a magnetic bead sorting were stimulated with OVA-pulsed bone marrow dendritic cells for 48 h and then re-stimulated with phorbol myristate acetate/ionomycin for up to 4 h. Cytokines in culture supernatants were quantified by ELISA. (**B**) Exposure to PM_2.5_ contributed to increased total IgE in the serum. Blood was collected from mice after 14 days of topical challenge with PM_2.5_ or the vehicle. Sera were isolated and measured for total IgE. Data are presented from three to four independent experiments as the mean ± SEM. * *p* < 0.05. ** *p* < 0.01.

Purified T cells were stimulated with BMDC in vitro to determine whether PM_2.5_ led to the immunological modulation of draining LNs. Cytokine secretions into the supernatants were quantified via ELISA. The levels of Th2 cytokines such as IL-4 (*p* < 0.001 versus vehicle) and IL-13 (*p* < 0.001 versus vehicle), but not the Th1 cytokine IFN-_Ɣ_, were significantly increased compared with those in the vehicle-treated mice (*p* = 0.217 versus vehicle; Figure 7A). PM-exposed groups also displayed significantly higher levels of serum IgE compared to the vehicle controls (*p* < 0.05 versus vehicle; Figure 7B).

## 4. Discussion

This study evaluated whether exposure to traffic-related PM_2.5_ modulates inflammatory responses on the ocular surface and in draining LNs in healthy mice. This PM was collected from the road dust of a downtown area near main streets in Gangdong-Gu, Seoul, Korea. Our study demonstrated that exposures to PM_2.5_ enhanced ocular surface impairment and allergic responses, as supported by increased apoptosis of the ocular surface, elevated levels of total IgE in the serum, and enriched secretions of Th2 cytokines in draining LNs. In addition, PM_2.5_ led to an increase in mast cell infiltration into the conjunctiva and maturation of DC to mediate the Th2 response. Our evidence suggests that traffic-related PM_2.5_ modifies and enhances the ocular allergic immune response in the mouse model, rather than dry eye disease (DED).

The ocular surface is exposed to the external environment and air pollution. Environmental pollution can cause ocular symptoms. Various investigations have demonstrated that exposure to higher levels of traffic-related air pollution increases ocular irritation and tear film instability, close to DED [4,9,10,11,17]. A high concentration of air pollution in metropolitan areas increases the likelihood of being diagnosed with dry eye disease three to four times, compared with relatively low concentrations of pollutants [14]. Gupta et al. demonstrated that air pollution was associated with a high prevalence of DED symptoms, such as irritation, the sensation of a foreign body, redness, tear film instability, and photophobia [26]. DED was recently defined as “a multifactorial disease of the ocular surface characterized by a loss of homeostasis of the tear film and accompanied by ocular symptoms” by the Tear Film & Ocular Surface Society (TFOS) Dry Eye Workshop II (DEW II) report [27]. DED presents as ocular surface discomfort, changes in visual acuity, and ocular surface damage and inflammation, including loss of goblet cells in the conjunctiva, aqueous tear deficiency, infiltration of immune cells into the cornea, and elevated levels of inflammatory cytokines in tears [28]. Torricelli et al. suggested a correlation between ambient levels of air pollution and tear osmolality, which is the core mechanism of ocular surface damage and inflammation in dry eye patients [9,17]. In this study, traffic-related PM_2.5_ increased corneal fluorescein staining and epithelial apoptosis, similarly to the clinical signs of DED. Our previous study on human corneal epithelial cells showed that traffic-related PM_2.5_ decreased cell viability and disrupted cellular membrane integrity [20]. 

According to the experimental DED studies, the stimulation and expansion of CD4+ T cells occur in the secondary lymphoid tissues in DED [29,30,31]. IFN-γ-secreting CD4+ T (Th1) and IL-17-secreting CD4+ T (Th17) cells are generated in the draining LNs of murine DED [29,30,31]. IFN-_Ɣ_ secreted from Th1 cells contributes to the corneal barrier disruption and decreased goblet cell density in the conjunctivae [32,33,34]. However, in our study, PM_2.5_ increased levels of Th2 cytokines such as IL-4 and IL-13 in the draining cervical LNs, but not of the Th1 cytokine IFN-_Ɣ_. Moreover, total serum IgE was significantly increased by PM_2.5_ exposures, like in allergic diseases. Allergic conjunctivitis, one of the most common ocular diseases, presents as an allergic inflammatory reaction to many substances. It is accompanied by discomfort, itching, conjunctival redness, swelling, and discharge. In this study, exposure to PM_2.5_ also presented as mild conjunctival redness and mucous discharge, but not significantly, compared to the vehicle control. Allergic conjunctivitis shares a common pathogenesis with other allergic diseases. Therefore, allergenic CD4+ T helper (Th)-2 cells and their cytokines (IL-4, IL-5, IL-13) mediate allergic responses, as supported by IgE production, mast cell and eosinophil accumulation, and mucus production [35]. 

Recently, some reports have indicated that air pollution causes allergic diseases. In a previous study in European cities, 15% of all childhood asthma exacerbations were attributed to exposure to road transport pollutants [36], and both urbanization and outdoor air pollution are important contributors to asthma [37,38]. Mimura et al. found a significant association between the number of outpatient visits for allergic conjunctivitis and the PM_2.5_ level, especially during the non-pollen season. Our findings were consistent with those of previous studies on the respiratory system [39]. Exposure to PM_2.5_ is associated with asthma and allergic respiratory symptoms [39,40], but little is known about the influence of PM_2.5_ on ocular allergies. Several experimental studies demonstrated that combined exposure to diesel exhaust particles and an antigen from a house dust mite induced a mixed Th2 and Th17 response in a murine model, and exposure to PM results in oxidative injury to the airways, inflammation, remodeling, and increased risk of aeroallergen sensitization [39,40,41]. In this study, traffic-related PM_2.5_ increased the maturation of APCs at cervical draining LNs, which might be mediated by increased inflammatory cytokines, including IL-1β, IL-6, and TNF-α, at the ocular surface. Inflammatory signals such as TNF, IL-1β, and heat shock proteins increase DC maturation (increased expression of CD80 and MHCII), initiating a T-cell-mediated immune response, as reported previously [42,43]. These studies have shown that a PM-mediated enhanced activation of antigen-presenting cells (APCs) such as DC could augment the adaptive immune system, including proliferation of Th2 cells, Th2 cytokine secretion, mast cell and eosinophil recruitment, and severe allergic inflammation [43,44]. Our data also showed increased maturation of APCs at cervical LNs. We postulate that maturation of APCs mediated by PM_2.5_ enhances the Th2 response in draining LNs. PM_2.5_ could drive a pro-allergic Th2-dominant immune response, orchestrated by DC [43,44,45]. Although the mechanisms by which traffic-related PM_2.5_ modulates ocular allergic inflammation remain unclear, we suggest they include PM-mediated ocular surface inflammation, APC maturation, aeroallergen sensitization, and Th2-derived immunological responses [43,44,45,46].

One of the hallmarks of DED is a decrease in the number of goblet cells [33,47]. However, our results indicate that hyperplasia or proliferation of goblet cells in the conjunctiva was not significantly changed by PM_2.5_, but mRNA expression of MUC5AC was increased after PM_2.5_ exposure. Previous investigations have reported that higher levels of traffic-related air pollution increased the frequency of ocular irritation with goblet cells hyperplasia in tarsal conjunctiva [9]. The goblet cell hyperplasia and increased mucin production represent an ocular mucosa response to chronic inflammatory or toxic stimuli [48,49]. Kondo et al. demonstrated that human recombinant IL-13, but not IL-4, can differentiate mature goblet cells that produce MUC5AC proteins in in vitro guinea pig tracheal epithelial cells [50]. Increased levels of IL-13, according to the Th2 response after traffic-related PM_2.5_ exposure, might stimulate goblet cell differentiation and MUC5AC production at the conjunctivae, which is a common pathogenesis in allergic diseases. Moreover, goblet cell hyperplasia with mucin production seems to protect the ocular surface from environmental toxins [17]. Research has demonstrated the major signs and symptoms of air pollution are similar to those of DED, including ocular fluorescein staining, epithelial apoptosis, elevated tear osmolality, loss of goblet cells, and ocular irritation, which may occur as a result of chronic exposure to air pollution [14,17].

One major limitation of this study is that the environmental exposure to PM_2.5_ might be different from the topical administration of PM_2.5_. We need to modify the concentration and the mode of exposure in future studies. In addition, a human ocular surface might be different from an animal model. Therefore, a large-scale, long-term clinical study is needed to evaluate the actual effects of fine ambient PM exposure on the ocular surface.

## 5. Conclusions

Traffic-related PM_2.5_ exposure presented Th2-dominant allergic immune response in the draining LNs, rather than the Th1 response of the DED model. PM_2.5_ increased epithelial disruption and inflammation on the ocular surface, a main sign of DED, which might increase the penetration of aeroallergens into the ocular tissues and maturation of APCs to develop ocular allergy. This research may help diagnose and treat air-pollution-associated ocular disease.
